# Influenza Illness among Case-Patients Hospitalized for Suspected Dengue, El Salvador, 2012

**DOI:** 10.1371/journal.pone.0140890

**Published:** 2015-10-20

**Authors:** Rafael Chacon, Alexey Wilfrido Clara, Jorge Jara, Julio Armero, Celina Lozano, Nathalie El Omeiri, Marc-Alain Widdowson, Eduardo Azziz-Baumgartner

**Affiliations:** 1 Influenza Unit, University of the Valley of Guatemala, Guatemala City, Guatemala; 2 Influenza Program, Centers for Disease Control and Prevention for Central American Region, Guatemala City, Guatemala; 3 Health Surveillance Directorate, Ministry of Health of El Salvador, San Salvador, El Salvador; 4 National Influenza Center, Ministry of Health of El Salvador, San Salvador, El Salvador; 5 Influenza Unit, Training Programs in Epidemiology and Public Health Interventions Network, Guatemala City, Guatemala; 6 Influenza Division, National Center for Immunization and Respiratory Diseases, Centers for Disease Control and Prevention, Atlanta, Georgia, United States of America; University Hospital San Giovanni Battista di Torino, ITALY

## Abstract

We estimate the proportion of patients hospitalized for suspected dengue that tested positive for influenza virus in El Salvador during the 2012 influenza season. We tested specimens from 321 hospitalized patients: 198 patients with SARI and 123 patients with suspected dengue. Among 121 hospitalized suspected dengue (two co-infected excluded) patients, 28% tested positive for dengue and 19% positive for influenza; among 35 with suspected dengue and respiratory symptoms, 14% were positive for dengue and 39% positive for influenza. One percent presented co-infection between influenza and dengue. Clinicians should consider the diagnosis of influenza among patients with suspected dengue during the influenza season.

## Introduction

Influenza and dengue are diseases of international public health importance because of their associated morbidity and mortality [1]. In tropical regions like Central America, outbreaks of seasonal influenza and dengue occur annually from June through September [2,3]. The similar clinical presentation of patients infected with influenza or dengue makes differential diagnosis difficult [4,5] and may delay appropriate antiviral treatment of persons with influenza [6]. This differentiation is especially challenging when laboratory tests to detect these viruses are not readily accessible to support timely therapeutic decisions.

During 2009, the emergence of the pandemic influenza A(H1N1) virus coincided with dengue outbreaks in several countries (S1 Appendix). Physicians in Central America obtain specimens for influenza testing from patients hospitalized with severe acute respiratory infection (SARI) [7], but infrequently from patients hospitalized with suspected dengue. Physicians may consider the presence of influenza between suspected dengue cases; this depends on their experience. The criteria for deciding to test for influenza in suspected dengue cases are not included in the guidelines of these countries [8].

In this study, we estimate the proportion of patients hospitalized for suspected dengue tested positive for influenza virus in El Salvador during the 2012 influenza season. We also compare the respiratory symptoms among persons with dengue or influenza illnesses in order to explore the capability of case definitions for SARI and suspected dengue for detect cases related to influenza and dengue viruses.

## Materials and Methods

**Fig 1:**
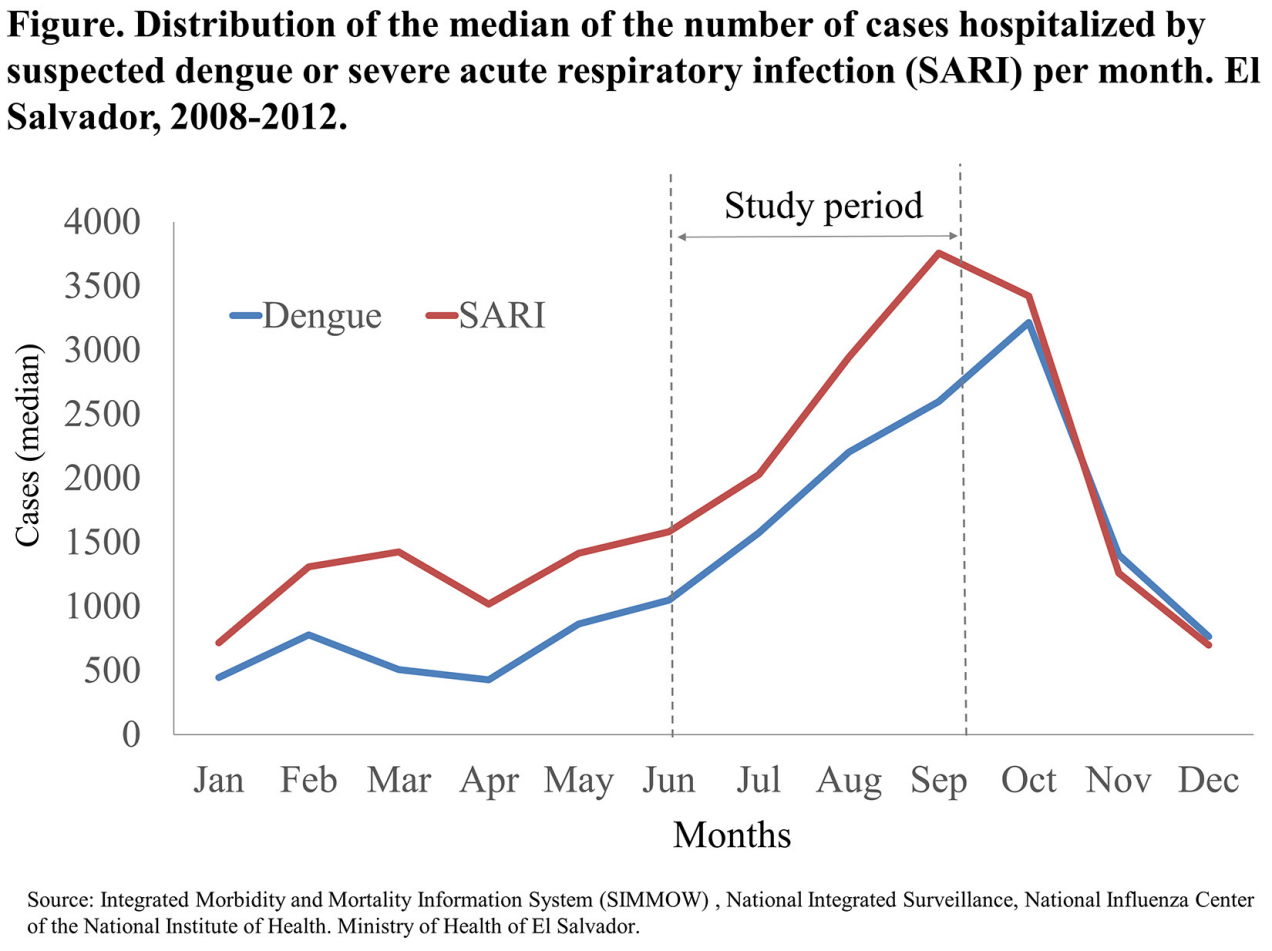
Trend.

We analyzed data from a cross-sectional study originally designed to estimate the prevalence of co-infection with influenza and dengue among hospitalized patients in El Salvador during the 2012. We conducted the study from July 1 to September 30, 2012, coinciding with the period that historically has the highest influenza and dengue activity in El Salvador (Fig 1) [9].

We enrolled hospitalized patients with SARI and/or suspected dengue at three reference hospitals and one general hospital that comprised the influenza surveillance network in El Salvador. We solicited to all adults potential participants (≥ 18 years) their written consent to participate in the study. If they were not able to speak because of medical condition, we asked for written consent to next of kin. If this participant regained their cognitive faculties, was performed the consent process; this was documented in the appropriate form. All potential participants children (<18 years) were asked the written consent of their parents or guardians. In addition to informed consent, to children ≥ 7 years old who were able to give assent, we read the Assent Form, and if they were agree, we asked to sign it. In all cases, we gave a hard copy of the Informed Consent Form. Consent procedures were approved by the Ethics Committee for Clinical Research of El Salvador, and the Committee on Ethics in Research of the Universidad del Valle de Guatemala.

We identified patients using the WHO SARI case definition [7], and the El Salvador Ministry of Health case definition for dengue (adapted from WHO) [10] (Table 1).

**Table 1:** Case definitions used for the study.

Severe acute respiratory infection (SARI) case-definition:	Suspected dengue case-definition:	Criteria for severe dengue:
Sudden onset of fever over 38° C and Cough or sore throat, and dyspnea, and requiring hospitalization.	**Sudden onset of fever, and at least two of the following manifestations**: Malaise, Headache, Retro ocular pain, Myalgia / arthralgia, Rash, Anorexia, Nausea, History of spontaneous bleeding, Tourniquet test positive, Leukopenia.	Severe plasma leakage, or Severe bleeding, or Severe organ involvement.
	**Or any of the warning signs**: Sustained abdominal pain, Intense vomiting, Body fluid accumulation, Mucosal bleeding, Lethargy / irritability, Asthenia, Enlarged liver (greater than 2 cm), Increased hematocrit associated with a rapid decrease of platelet count within a 24 hour period).	

We excluded newborns (unlikely to present with classical influenza symptoms) [11], cases hospitalized after five days of symptoms onset because a lower probability of accurate laboratory confirmation for dengue using rtPCR [12], hospital-acquired infections, and persons admitted for social reasons (e.g. inability to treat mild illnesses at home because of their living situation).

To ascertain dengue or SARI case status, we collected clinical data through interviews with patients or their proxy (i.e., parents or guardians), clinical examinations, and medical record reviews. We considered cough, rhinorrhea, nasal congestion, sneezing, inspiratory crackles, sore throat, wheezing and shortness of breath as respiratory symptoms. We classified cases as very severe if they were hospitalized beyond five days; required restricted antibiotics (drugs for resistant bacteria) [13], mechanical ventilation, or intensive care; or died during their hospitalization. At enrollment, we obtained nasal and pharyngeal swab and blood samples from all participants. Specimens were tested at the National Reference Laboratory for influenza and dengue viruses through real time reverse transcription polymerase chain reaction (rtPCR). We defined thrombocytopenia as <150,000 platelets per mm3 [12].

Detection of influenza and dengue virus using rtPCR were performed using the CDC protocols. We identified influenza subtypes: A/H1, A/H3, 2009 AH1N1pdm and B; and dengue serotypes 1, 2, 3, and 4. Extraction kit Ag-Path (Ambion) and RNA purification Qiagen Qia-amp, and Invitrogen PureLink wer used. The spin column and QIAcube methods were used. The sensitivity of the method for detection of influenza virus was 99.3% and 87.1% for dengue. The specificity for influenza virus was 92.3% and 97.7% for dengue (data are theoretical sensitivity and specificity of the method) [14,15].

We assessed the distribution of respiratory symptomatology among patients with laboratory-confirmed dengue or influenza through PCR.

## Results

We identified 392 cases that met case definition for SARI or suspected dengue, of which 321 accepted to participate (82%) and 71 refused to participate (18%). We tested specimens from 321 hospitalized patients: 198 patients with SARI and 123 patients with suspected dengue. One hundred and sixty-two (50%) were female and 46 patients tested positive for influenza. SARI cases were younger (median age 2 years; IQR 1–7) than suspected dengue cases (median age 11 years; IQR 6–14) (p<0.01, Mann-Whitney).

Excluding three cases with influenza-dengue co-infection, tested positive for influenza 10% of SARI cases (20 of 198 cases) and 19% of suspected dengue cases (23 123 cases) (Table 2). Thirty nine percent (14 of 36 cases) of suspected cases of dengue were positive for influenza, had some respiratory symptoms, but not required to meet the case definition for SARI (always excluding cases of co-infection). Twenty-five (54%) of the 46 patients who tested positive for influenza and negative for dengue were hospitalized as severe suspected dengue cases. Excluding cases with co-infection, of the 43 cases tested positive for influenza, 53% (23 cases) were hospitalized for suspected dengue.

**Table 2:** Laboratory test results for influenza and dengue viruses in patients with suspected dengue or severe acute respiratory illness (SARI) at hospital admission, 4 hospitals in El Salvador, during July 1 through September 30, 2012.

	Influenza (+)		Influenza (−)	
	Dengue (+) (Co-infection)	Dengue (−)	Dengue (+)	Dengue (−)
**SARI** (n = 198)	1 (0.5%, 95%CI 0−2%)	20 (10%, 95%CI 6−14%)	4 (2%, 95%CI 0−4%)	173 (88%, 95%CI 83−92%)
**Suspected dengue**(n = 123)	2 (2%, 95%CI 0−4%)	23 (19%, 95%CI 12−26%)	34 (28%, 95%CI 20−36%)	64 (52%, 95%CI 43−61%)
Suspected dengue with respiratory symptoms (n = 36)	1 (3%, CI95% 0–15%)	14* (39%, CI95% 22–56%)	5 (14%, CI95% 5–29%)	16 (44%, CI95% 27–62%)
Suspected dengue without respiratory symptoms (n = 87)	1 (1%, CI95% 0–6%)	9 (10%, CI95% 3–17%)	29 (33%, CI95% 23–44%)	48 (55%, CI95% 44–66%)
**Total** (N = 321)	3 (1%,95%CI 0.2–3%)	43 (13%,95%CI 10–17%)	38 (12%,95%CI 8–16%)	237 (74%, 95%CI 69–79%)

*One (7%) of 14 suspected dengue case-patients tested positive for influenza but negative for dengue; this case also met the SARI case definition.

Respiratory symptoms were present in some suspect dengue cases. Thirty-six (29%) of the 123 patients with suspected dengue developed at least one respiratory symptom, but did not meet the current SARI case definition (no dyspnea) (Table 1). While 14 (39%) of 36 suspected dengue cases with respiratory symptoms tested positive for influenza (and dengue negative), only 9 (10%) of 87 suspect dengue case-patients without respiratory symptoms (and dengue negative) tested positive for influenza. One (7%) of the 14 suspected dengue case-patients with respiratory symptoms tested positive for influenza (and negative for dengue) and met the SARI case-definition.

We found that of the 23 suspected dengue cases tested positive for influenza, the most prevalent respiratory symptoms were cough with 43% (10 cases), rhinorrhea 35% (8 cases) and nasal congestion 35% (8 cases).

Among hospitalized patients with suspected dengue who tested positive for influenza (and negative for dengue), 61% (14 of 23 cases) presented with respiratory symptoms. Cough (43%), rhinorrhea (35%) and nasal congestion (35%) were the most prevalent among these cases. Only one case had shortness of breath (Table 3).

**Table 3:** Respiratory symptoms among hospitalized patients with suspected dengue, 4 hospitals in El Salvador, during July 1 through September 30, 2012.

Respiratory symptomatology	Influenza (+)* (n = 23)	Dengue (+)* (n = 34)	Negatives (n = 64)
	Cases (%)	Cases	Cases
Cough	10 (43)	5 (15)	13 (20)
Rhinorrhea	8 (35)	2 (6)	5 (8)
Nasal congestion	8 (35)	4 (12)	6 (9)
Sneezing	7 (30)	3 (9)	5 (8)
Inspiratory crackles	4 (17)	4 (12)	1 (2)
Sore throat	3 (13)	0	8 (13)
Wheezing	2 (9)	1 (3)	3 (5)
Shortness of breath	1 (4)	4 (12)	3 (5)
**Any respiratory symptomatology**	**14 (61)**	**5 (15)**	**16 (25)**

* Positive by rtPCR. Coinfection cases not included in the table.

Among those who tested negative for influenza, 28% of suspected dengue and 2% of SARI case-patients tested positive for dengue. Among all cases, only 3 (1%) tested positive for both influenza and dengue (Table 1). Forty-four percent (19 cases) of 43 influenza-positive and 37% (14 cases) of 38 dengue-positive had severe illness.

Of the 46 influenza-rtPCR positive cases, 44 had influenza B and 2 had influenza A(H1N1)pnd09; 11 (26%) had thrombocytopenia.

## Discussion

Our results suggest that hospitalized persons with influenza illness are often misdiagnosed as having dengue when influenza and dengue epidemics coincide. Most case-patients with suspected dengue that tested positive for influenza (and negative for dengue) presented respiratory symptoms (61%). In addition, one quarter of influenza case-patients had thrombocytopenia, a finding clinicians typically associate with dengue [10,12]. Only 10% of suspected dengue case-patients without respiratory symptoms, however, tested positive for influenza. Clinicians should consider the possibility of influenza illness among suspected dengue case patients during the influenza season, particularly if these have respiratory symptoms and/or meet the SARI case definition.

Public health officials should anticipate that a significant proportion of hospitalized patients with influenza illness may be clinically misdiagnosed with dengue infection. Surveillance platforms from which physicians infrequently obtain respiratory samples from suspected dengue patients may systematically underestimate influenza activity.

The most recent SARI case definition proposed by WHO is: acute respiratory infection with history of fever and cough within 10 days of symptoms among persons requiring hospitalization [16]. In countries where influenza and dengue co-circulate, the new WHO SARI case definition may increase the number of influenza-positives identified by surveillance platforms (because dyspnea is not included in this case definition).

We found a higher prevalence of cough, rhinorrhea and nasal congestion, in patients hospitalized for suspected dengue tested positive for influenza; this respiratory symptomatology could guide to test for influenza in those cases hospitalized for suspected dengue. This sign is described in the literature to differentiate influenza other acute febrile infections [17]. As has been described in the literature, the tourniquet test can be useful to differentiate dengue from other acute febrile illnesses [18].

Our study had some limitations. We performed the study during an influenza season in which influenza B virus was predominant in El Salvador. Some physicians may have classified case-patients as SARI or suspected dengue on admission based on their clinical impression rather than on the strict application of case-definition criteria. We sampled suspected dengue case-patients if they presented within five days of symptoms onset and may have missed dengue case-patients who presented thereafter.

## Conclusions

In our study, while dengue was rarely identified among SARI case-patients, influenza was often identified in those with suspected dengue, particularly if these had respiratory symptoms. Clinicians should consider influenza in the differential diagnosis among these patients during the influenza season, even if these present with thrombocytopenia. Surveillance staff should also consider obtaining respiratory specimens from suspected severe dengue case-patients who also meet the new WHO SARI case definition.

## Supporting Information

S1 Appendix Previous studies that found coinfection between influenza and dengue. (DOCX)

S1 Database Database of the study. (DTA)
