# Supplementary material for: Influenza Illness among Case-Patients Hospitalized for Suspected Dengue, El Salvador, 2012
Source: PLoS One. 2015 Oct 20;10(10):e0140890. doi: 10.1371/journal.pone.0140890 (PMC4618691; doi:10.1371/journal.pone.0140890)
Supplement: S1 Appendix — (DOCX) [file pone.0140890.s001.docx]

| **S1 Appendix 1. Previous studies that found coinfection between influenza and dengue.** | | | | | | | | |
| --- | --- | --- | --- | --- | --- | --- | --- | --- |
| **Epidemiologic situation / country/Year [Ref]** | **Reported cases** | **Age** | **Symptomathology ^a^** | |  | **Lab. Diagnosis** | | **Healt care level and outcome** |
|  |  |  | **Acute reapiratory ilness** | **Dengue** |  | **Influenza** | **Dengue** |  |
| **Seasonal Flu** |  |  |  |  |  |  |  |  |
| Thailand (2001-2002) ^[1]^ | 4 | 15-89 years old | NR | NR |  | ELISA^c^ | ELISA^c^ | Admitted in hospital |
| Thailand (1991-1993) ^[2]^ | 1 | 62 years^b^ | NR | NR |  | ELISA^c^ | ELISA^c^ | Deceased |
| **Pandemic flu (H1N1pnd09)** |  |  |  |  |  |  |  |  |
| Saudi Arabia ^[3]^ | 1 | 44 Years old | Yes | No |  | PCR | PCR | Admitted in ICU |
| Puerto Rico ^[4]^ | 1^d^ | 33 | Yes | No |  | PCR | PCR | Outpatient case |
| Puerto Rico  ^[5]^ | 1 | NR | NR | NR |  | PCR | NR | NR |
| Vietnam ^[6]^ | 2 | NR | NR | NR |  | NR | NR | Deceased |
| El Salvador ^[7]^ | 4 | Two children | NR | NR |  | PCR | ELISA^e^ | -- |
| India ^[8]^ | 1 | 23 Years old | Yes | Yes |  | ELISA^c^ | ELISA^c^ | Admitted in ICU |
| Barbados ^[9]^ | 8 | NR | NR | NR |  | PCR | PCR^f^ | -- |
| Nicaragua ^[10]^ | 4 | 5-11 Years old | 3 yes | 4 yes |  | PCR | PCR^f^ | 3 admitted in ICU,  1 deceased |
| ^a^ Characteristic symptomas for each disease, excluded fever. ^b^ Also presented diagnosis of tiphoidea fever. ^C^ Paired samples. ^d^ Health care worker. ^e^  Single sample by case. ^f^ Dengue 3. NR Not reported. ICU Intensive Care Unit. | | | | | | | | |
| **References** | | | | | | | | |
| 1. Suttinont C, Losuwanaluk K, Niwatayakul K, Hoontrakul S, Intaranongpai W, Silpasakorn S, et al. Causes of acute, undifferentiated, febrile illness in rural Thailand: results of a prospective observational study. Ann Trop Med Parasitol. 2006;100:363–70. DOI: 10.1179/136485906X112158 | | | | | | | | |
| 2. Leelarasamee A, Chupaprawan C, Chenchittikul M, Udompanthurat S. Etiologi of acute undifferentiated febrile illness in Thailand. J Med Assoc Thai.2004;87:464–72. | | | | | | | | |
| 3. Hussain R, Al-Omar I, Memish ZA. The diagnostic challenge of pandemic H1N1 2009 virus in a dengue-endemic region: a case report of combined infection in Jeddah, Kingdom of Saudi Arabia. Journal of infection and public health [Internet]. 2012 Apr [cited 2013 Apr 3];5(2):199–202. Available from: http://www.ncbi.nlm.nih.gov/pubmed/22541269 | | | | | | | | |
| 4. Rodriguez LE, Tomashek KM, Gregory CJ, Munoz J, Hunsperger E, Lorenzi OD, et al. Co-infection with dengue virus and pandemic (H1N1) virus [letter]. Emerg Infect Dis [serial on the Internet]. 2010 May [date cited]. http://wwwnc.cdc.gov/eid/article/16/5/09-1920.htm | | | | | | | | |
| 5. Lorenzi OD, Gregory CJ, Santiago LM, Acosta H, Galarza IE, Hunsperger E, et al. Acute febrile illness surveillance in a tertiary hospital emergency department: comparison of influenza and dengue virus infections. The American journal of tropical medicine and hygiene [Internet]. 2013 Mar [cited 2013 Jul 12];88(3):472–80. Available from: http://www.ncbi.nlm.nih.gov/pubmed/23382160 | | | | | | | | |
| 6. Tre T. Influenza Pandemic (H1N1) 2009 (96). Thanh Nien Daily News. Published in Nov 12, 2009. Available in: http://www.thanhniennews.com/healthy/?catid=8&newsid=53607 | | | | | | | | |
| 7. Martinez L. Pacientes con Dengue y H1N1. La Prensa Grafica. 24 de Octubre de 2009; Mediacenter [Accesado 15 de Abril 2012]. Disponible en: http://www.laprensagrafica.com/lo-del-dia-edi/68270-pacientes-con-dengue-y | | | | | | | | |
| 8. Borthakur B, Panwar D, Garg R, Pawar M. Viral co-infection with dengue and H1N1 virus in a critical care setting. J Anaesthesiol Clin Pharmacol. 2011 Apr-Jun; 27(2): 236–238. | | | | | | | | |
| 9. Pan American Health Organization. Pandemic (H1N1) 2009 Regional Update 03 Nov 2009. PAHO. Available in: http://new.paho.org/hq/dmdocuments/2009/RU_Pandemic_H1N1_2009_November3_2009.pdf | | | | | | | | |
| 10. Perez MA, Gordon A, Sanchez F, Narvaez F, Gutierrez G, Ortega O, Nuñez A, Harris E, Balmaseda A. Severe coinfections of dengue and pandemic influenza A H1N1 viruses. Pediatr Infect Dis J. 2010 Nov;29(11):1052-5. | | | | | | | | |
